# A Facile Method to Produce N-Terminally Truncated α-Synuclein

**DOI:** 10.3389/fnins.2022.881480

**Published:** 2022-05-27

**Authors:** Rebecca J. Thrush, Devkee M. Vadukul, Francesco A. Aprile

**Affiliations:** ^1^Department of Chemistry, Molecular Sciences Research Hub, Imperial College London, London, United Kingdom; ^2^Institute of Chemical Biology, Molecular Sciences Research Hub, Imperial College London, London, United Kingdom

**Keywords:** α-synuclein, Parkinson’s disease, amyloid fibrils, post-translational modification, N-terminal truncation

## Abstract

α-Synuclein is a key protein of the nervous system, which regulates the release and recycling of neurotransmitters in the synapses. It is also involved in several neurodegenerative conditions, including Parkinson’s disease and Multiple System Atrophy, where it forms toxic aggregates. The N-terminus of α-synuclein is of particular interest as it has been linked to both the physiological and pathological functions of the protein and undergoes post-translational modification. One such modification, N-terminal truncation, affects the aggregation propensity of the protein *in vitro* and is also found in aggregates from patients’ brains. To date, our understanding of the role of this modification has been limited by the many challenges of introducing biologically relevant N-terminal truncations with no overhanging starting methionine. Here, we present a method to produce N-terminally truncated variants of α-synuclein that do not carry extra terminal residues. We show that our method can generate highly pure protein to facilitate the study of this modification and its role in physiology and disease. Thanks to this method, we have determined that the first six residues of α-synuclein play an important role in the formation of the amyloids.

## Introduction

α-Synuclein (α-syn) is a 14 kDa intrinsically disordered protein that predominantly localizes at the presynaptic terminals of neurons (Maroteaux et al., 1988; Weinreb et al., 1996). There, it is normally responsible for the recycling of neurotransmitter vesicles, mediating neurotransmitter storage and release (Cheng et al., 2011; Burré et al., 2014; Duce et al., 2017). In Parkinson’s disease (PD) and several other forms of neurodegeneration, this protein forms characteristic fibrillar aggregates, the amyloids, that are the major components of proteinaceous inclusions in the brain called Lewy Bodies (LBs) (Spillantini et al., 1997; Spillantini et al., 1998). α-Syn comprises of three domains: an amphipathic N-terminal tract (residues 1–60) with α-helical propensity, an aggregation-prone central region (residues 61–95) called Non-Amyloid-β Component (NAC) that forms the core of the amyloid fibrils, and a negatively charged C-terminal region (residues 96–140) that protects from aggregation, binds to metal ions, such as calcium, and also mediates the interaction with other amyloidogenic proteins (Burré et al., 2018; Lautenschläger et al., 2018; Dasari et al., 2019). The N-terminal tract is crucial for several important functions of α-syn (Lee et al., 2002; Cheng et al., 2011; Burré et al., 2014; Lorenzen et al., 2014; Duce et al., 2017; McGlinchey et al., 2021). In fact, although all domains play a role in the association of the protein to lipid membranes, the N-terminus is the primary domain responsible for this interaction, which, under normal conditions, promotes the fusion of neurotransmitter vesicles to the presynaptic button (Burré et al., 2014; Fusco et al., 2014; Lautenschläger et al., 2018). In neurodegeneration, this interaction triggers the amyloid aggregation of the protein (Lee et al., 2002; Galvagnion et al., 2016). Furthermore, it has been proposed that the N-terminal tract can establish long-range electrostatic interactions with the C-terminal region of α-syn (Bernadó et al., 2005; Bertoncini et al., 2005; Dedmon et al., 2005; Stephens et al., 2020), leading to more compact protein conformations (Dedmon et al., 2005; Cho et al., 2009) and shielding the NAC region from aggregation (Stephens et al., 2020). It has also been proposed that alterations to these interactions may be driving α-syn liquid-liquid phase separation (LLPS) (Hardenberg et al., 2020; Ray et al., 2020; Sawner et al., 2021).

α-Syn undergoes several post-translational modifications (PTMs) that affect both its normal and pathological functions (Anderson et al., 2006). In particular, the protein is N-terminally truncated into fragments of different lengths *in vitro* (Vlad et al., 2011; Vlad et al., 2012) and *in vivo* (Liu et al., 2005; Dufty et al., 2007; Kellie et al., 2014; Killinger et al., 2018). It has been proposed that α-syn N-terminal truncation may be a consequence of auto-proteolytic cleavage, protease cleavage and/or incomplete digestion by the proteosome (Liu et al., 2005; Dufty et al., 2007; Vlad et al., 2011; Killinger et al., 2018). Some of these fragments have been reported to have different aggregation mechanisms and form structurally polymorphic amyloids with respect to the wild-type (WT) protein (McGlinchey et al., 2021). Nevertheless, we still lack important details regarding the mechanisms of aggregation of α-syn N-terminally truncated fragments, because of challenges associated with the recombinant production of these proteins. In fact, currently, most of these variants are produced with an additional starting Met. The presence of the extra Met allows for facile recombinant expression in *Escherichia coli* (*E. coli*). Additionally, these protein variants have contributed to unveil how α-syn domains regulate the function and aggregation of the protein (Vamvaca et al., 2009; Lorenzen et al., 2014). On the other hand, the extra Met may also affect the binding to membranes and aggregation mechanisms, which depend on just a few (∼10–20) N-terminal residues (Fusco et al., 2014; Kumari et al., 2021; McGlinchey et al., 2021; Yang et al., 2021). To date, the only native N-terminal truncations to be recombinantly produced are those starting with a Gly at positions 14, 36, and 41 and have been generated with an *ad hoc* protocol based on placing the Gly downstream of the cleavage site for the tobacco etch virus (TEV) (McGlinchey et al., 2021). To expand the repertoire of α-syn N-terminal truncations that can be produced recombinantly, here, we described an intein-based protocol (Chong et al., 1998) to generate N-terminally truncated variants of α-syn with no overhanging residues. A key advantage of our method is that it does not require any proteases (e.g., TEV), making it time effective. Inteins have already successfully been used as purification tags (Uversky et al., 2001; Batjargal et al., 2015) and in chemical ligation protocols (Haney et al., 2016) of α-syn, proving this a robust purification strategy. In this paper, we describe a novel strategy to use inteins to introduce N-terminal truncations into α-syn. As a proof-of-principle, we show the efficacy of our method in producing the N-terminally truncated variant of α-syn that lacks the first 6 residues (7–140 α-syn) (Vlad et al., 2011).

## Materials and Methods

### Fusion Protein Expression and Purification

The intein-7–140 α-syn fusion protein cDNA was synthesized by GenScript and inserted into a pT7-7 plasmid. This plasmid will hereafter be referred to as pT7-7 Int7-140αSyn. BL21-Gold (DE3) competent *E. coli* cells (Agilent Technologies, Santa Clara, CA, United States) were transformed with the pT7-7 Int7-140αSyn plasmid, and the cells grown in 2 L of Overnight Express Instant LB medium (Merck, Darmstadt, Germany) containing 100 μg/ml ampicillin at 28°C for approximately 32 h. The cells were harvested by centrifugation and resuspended in 100 ml of ice-cold column buffer [20 mM 4-(2-hydroxyethyl)-1-piperazineethanesulfonic acid (HEPES), 0.5 M NaCl, 1 mM ethylenediaminetetraacetic acid (EDTA), pH 8.5] supplemented with EDTA-free protease inhibitors (Roche, Basel, Switzerland). 0.2% Tween 20 was also added to reduce non-specific protein binding to the chitin resin. Cell lysis was induced by sonication on ice. Following centrifugation and syringe filtration, gravity chromatography was used to cleave and purify 7–140 α-syn (Chong et al., 1998). To do so, the cell lysate was loaded on a chitin column equilibrated in column buffer. Unbound proteins were removed by washing the resin with 5 column volumes (CVs) of column buffer. The column was then equilibrated with 3 CVs of cleavage buffer [20 mM HEPES, 0.5 M NaCl, 1 mM EDTA, 50 mM β-mercaptoethanol (BME), pH 8.5] before the flow was stopped and the column incubated at room temperature for 44 h to facilitate cleavage. The target protein was eluted in 2 ml fractions using the column buffer. Fractions expected to contain α-syn (determined by absorbance at 275 nm using ε_275 *nm*_ = 5600 M^–1^cm^–1^) were combined and dialyzed overnight in PBS, pH 7.4 to remove the maltose binding protein (MBP) fragment and excess BME. The dialyzed sample was purified further by size-exclusion chromatography (SEC) using a HiLoad 16/600 Superdex 75 pg column (GE Healthcare, Chicago, IL, United States). Final protein concentration was determined by absorbance at 275 nm using ε_275 *nm*_ = 5600 M^–1^cm^–1^ as measured by UV-Vis spectrophotometry. Samples were taken throughout expression and purification for analysis by sodium dodecyl sulfate–polyacrylamide gel electrophoresis (SDS-PAGE) to determine the success and efficiency of each step.

### Expression and Purification of Wild-Type α-Synuclein

pT7-7 WT α-syn construct [Addgene, Watertown, NY, United States, gifted from Hilal Lashuel (Paleologou et al., 2008)], was transformed into BL21-Gold (DE3) competent *E. coli* cells and WT α-syn expressed according to the manufacturer’s instructions. Expression was scaled up to 1–5 L as desired and carried out at 28°C overnight. The cells were harvested by centrifugation and resuspended in 20 mM Tris–HCl, pH 8.0 including 1 mM EDTA and protease inhibitors. WT α-syn was then purified as previously described (Arter et al., 2020).

### Electrospray Ionization Mass Spectrometry

Purified protein samples (∼20 μM) in HPLC grade water were analyzed by electrospray ionization mass spectrometry (ESI-MS) to confirm molecular weight and sample purity. ESI-MS was performed by Lisa Haigh using the Chemistry Mass Spectrometry facilities available at the Molecular Sciences Research Hub, Department of Chemistry, Imperial College London. The data were plotted using GraphPad Prism version 9.3.1 for Windows (GraphPad Software, San Diego, CA, United States).

### Beaded Aggregation Assay

50 μM monomer solutions were aggregated in the presence of 20 μM Thioflavin T (ThT) and 0.02% NaN_3_. 170 μl of each sample (5 replicates) was loaded into a 96 well full-area plate (non-binding, clear bottomed, #655906, Greiner Bio-One, Frickenhausen, Austria) and incubated at 37°C for ∼140 h in a CLARIOstar Plus microplate reader (BMG Labtech, Ortenberg, Germany). Aggregation was promoted through linear shaking (300 rpm, 300 s before each cycle) with the addition of a single borosilicate bead (3 mm diameter) to each well. Fluorescent intensity measurements were taken using spiral averaging (5 mm diameter) using excitation 440 nm, dichroic 460 nm, and emission 480 nm filters, 3 gains and 50 flashes per well. The data were plotted using GraphPad Prism version 9.3.1 for Windows (GraphPad Software, San Diego, CA, United States). The reported *t*_1/2_ and *t*_*lag*_ values are averages of the independent replicates. The sigmoidal model provided by the software was used to estimate the *t*_1/2_ of aggregation. In particular, the *t*_1/2_ was estimated as the *x*-value at 50% of maximum fluorescence. The *t*_*lag*_ was estimated by extrapolation from the maximum aggregation rate.

### Analysis of Monomer Conversion and Transmission Electron Microscopy

The soluble and insoluble protein fractions after aggregation were separated by microcentrifugation (30 min, 16,900 *g*). Soluble fractions were then removed and analyzed by dot blot; 5 repeats of 5 μL aliquots were transferred to a nitrocellulose membrane which was then blocked before incubation with the anti-α-syn primary antibody MJFR1 (diluted 1:1000, Abcam, Cambridge, United Kingdom). The membrane was subsequently incubated with the goat anti-rabbit IgG (H + L) highly cross-adsorbed secondary antibody conjugated to the fluorophore Alexa Fluor plus 555 (diluted 1:5000, Thermo Fisher Scientific, Waltham, MA, United States) before imaging. The same dot blot protocol was used to analyze the reactivity of the antibody against the monomeric proteins. Mean gray values were measured using Fiji (Schindelin et al., 2012). Transmission electron microscopy (TEM) was performed to analyze fibril morphology at the endpoint of aggregation. To do so, the insoluble fibril pellets were resuspended in fresh PBS, pH 7.4 and re-centrifuged to remove any remaining soluble protein. The washed fibrils were then resuspended in 250 μL of PBS, pH 7.4 and 10 μL applied to carbon-coated copper 300 mesh grids. Following negative staining with 2% (w/v) uranyl acetate the fibrils were imaged using the Tecnai 12 Spirit transmission electron microscope [Thermo Fisher Scientific (FEI), Hillsboro, OR, United States] available at the Electron Microscopy Centre, Center of Structural Biology, Imperial College London. Fibril length and width was measured using Fiji (Schindelin et al., 2012). All data were plotted using GraphPad Prism version 9.3.1 for Windows (GraphPad Software, San Diego, CA, United States).

### Circular Dichroism

The far-UV Circular Dichroism (CD) spectra of monomer and insoluble fibril samples (∼20 μM) were taken using a Jasco J-715 (Jasco Applied Sciences, Halifax, NS, Canada). Spectra were taken between 200 and 250 nm, scanning speed 50 nm min^–1^ and 15 accumulations. A background spectrum of the sample buffer was subtracted from all sample spectra. Raw data (units mdeg) was converted to mean residue ellipticity (MRE, units deg cm^2^dmol^–1^) using (Greenfield, 2006):


(1) MRE=mdeg/(l*c*(n-1))

where mdeg is the raw data, n the number of amino acids, l the cuvette pathlength (mm), and c the protein concentration (M). The data were plotted using GraphPad Prism version 9.3.1 for Windows (GraphPad Software, San Diego, CA, United States).

### Fibril Digestion With Proteinase K

20 μM solutions of the insoluble protein fractions after aggregation were incubated with increasing concentrations of proteinase K (0, 5, 15, 30, and 50 μg/ml) at 37°C for 20 min. The samples were separated by SDS-PAGE before transfer to a nitrocellulose membrane. The membrane was then blocked, incubated with primary and secondary antibodies and imaged as described above. For each condition, the intensity of the band corresponding to monomeric α-syn was quantified using the software Fiji (Schindelin et al., 2012). The normalized data were plotted using GraphPad Prism version 9.3.1 for Windows (GraphPad Software, San Diego, CA, United States).

## Results and Discussion

### Experimental Design and Model Protein

**FIGURE 1:**
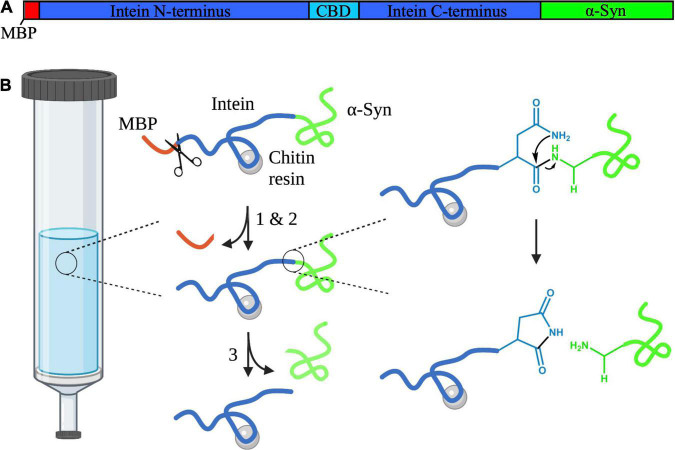
**(A)** Schematic diagram depicting the domains of the intein-7–140 α-syn fusion protein. The MPB fragment is shown in red, the Sce VMA intein containing the CBD in blue, and α-syn in green. This figure was created using SnapGene Viewer 6.0 (from Insightful Science; available at snapgene.com). **(B)** Representation of the VMA intein fusion protein cleavage reaction. (1) N-S acyl rearrangement at the intein N-terminus. (2) BME-mediated N-terminal cleavage. The BME is represented here as scissors. (3) Spontaneous cyclisation at the intein C-terminus occurs, cleaving truncated α-syn from the protein. This figure was created using BioRender and ChemDraw 18.2 (from PerkinElmer Informatics).

As a model protein for our study, we chose the N-terminally truncated variant 7–140 α-syn due to its possible role as a precursor for shorter α-syn fragments (Vlad et al., 2011). Additionally, similar fragments, such as those comprising of the regions 5–140 and 10–122 of α-syn, have been found in patient brains (Dufty et al., 2007; Kellie et al., 2014). To express this protein, we designed a vector based on the *Saccharomyces cerevisiae*’s vacuolar membrane ATPase (*Sce* VMA) intein (Chong et al., 1998). Inteins are protein segments that are removed from within larger precursors by protein splicing, followed by chemical ligation of the flanking regions, which are called exteins. It has been shown that, when the intein is inserted within unrelated proteins, its splicing activity is retained provided that the first residue of the C-terminal extein is either a Cys, a Ser, or a Thr (Cooper et al., 1993; Xu et al., 1993). Consequently, we engineered a construct, where the variant 7–140 α-syn was placed downstream of the *Sce* VMA intein. We also included a 10-residue fragment of the MBP at the N-terminus of the intein to facilitate expression, and the chitin binding domain (CBD) within the central region of the intein to enable affinity chromatography. It has been reported that insertion of the CBD does not affect the splicing activity of the VMA intein (Chong et al., 1998). In our system, the intein splicing occurs in three steps while the protein is bound to a chitin resin (Figure 1): (1) the spontaneous N-S acyl rearrangement of the intein N-terminal Cys forms a thioester intermediate with the C-terminal residue of the MBP fragment, (2) we expose the protein to BME, which attacks the thioester intermediate resulting in N-terminal cleavage and removal of the MBP fragment, and (3) a spontaneous cyclisation at the C-terminal Asn takes place, which cleaves the truncated α-syn from the intein.

### Expression and Purification of 7–140 α-Synuclein

To optimize the expression of the intein-7–140 α-syn fusion protein, we set up small scale cultures, where protein expression was induced with 1 mM IPTG at either 28°C overnight or 37°C for 4 h. Then, we analyzed the protein content of the cell extracts by SDS-PAGE. We found an enriched band of ∼70 kDa, which is the expected molecular weight of the fusion protein. This indicates that the protein is successfully expressed at both temperatures, with higher levels at 28°C (Supplementary Figure 1). Thus, we carried out our subsequent large-scale expression at 28°C overnight. Following our optimization, we were able to obtain a yield of approximately 1.5–2 mg/L of cell culture of highly pure 7–140 α-syn.

**FIGURE 2:**
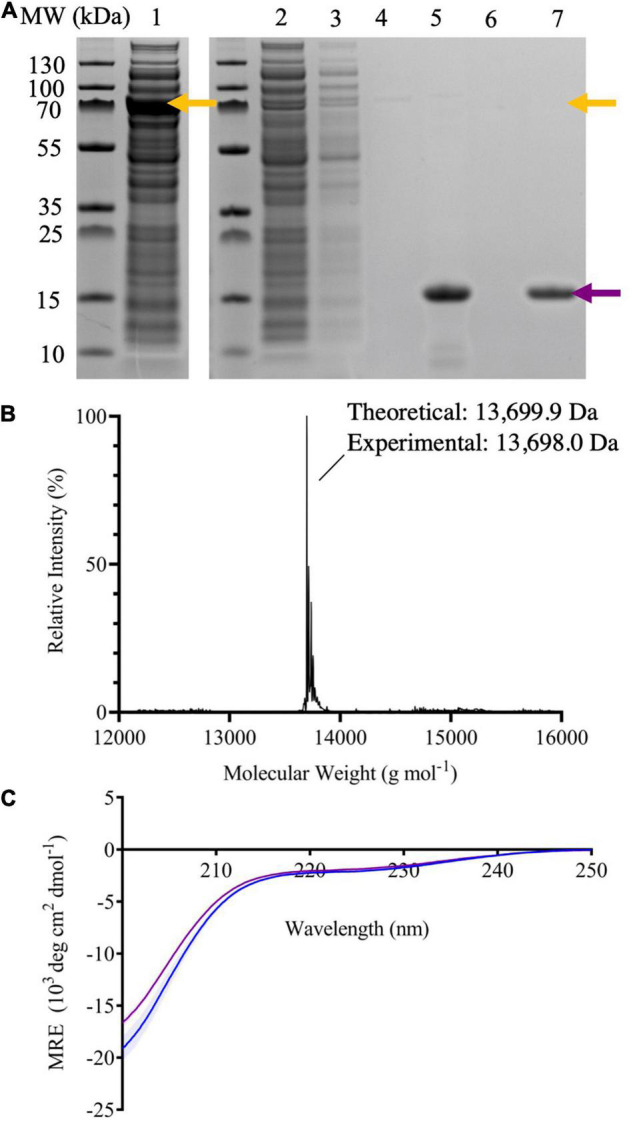
**(A)** SDS-PAGE of samples taken throughout the purification process of 7–140 α-syn; crude extract (lane 1), flow through after passing the crude extract through the column five times (lane 2), flow through after washing the column with column buffer (lane 3), flow through after equilibrating the column with cleavage buffer (lane 4), compiled fractions containing the target protein after dialysis (lane 5), flow through after stripping the column with NaOH (lane 6), and the target protein purified by SEC (lane 7). The yellow arrows indicate the molecular weight (MW) of the fusion protein, while the purple arrow the one of 7–140 α-syn. **(B)** The deconvoluted ESI-MS spectrum of 7–140 α-syn. Theoretical mass 13,699.9 Da, experimental mass 13,698.0 Da. **(C)** The CD spectra of monomeric WT α-syn (blue) and 7–140 α-syn (purple). *n* = 3, error bars representing the standard deviation are shown as transparent bars.

The purity of the protein was confirmed by ESI-MS and SDS-PAGE (Figures 2A,B). We compared the CD spectrum of monomeric 7–140 α-syn to that of WT α-syn (Figure 2C). Both spectra show a high content in random coil, which is in agreement with the disordered conformation of the monomeric protein. We also analyzed the purified sample by native PAGE before and after boiling at 80°C for 20 min, reproducing the heat precipitation step used in WT α-syn purification (Supplementary Figure 2). 7–140 α-Syn ran in a similar manner to the WT protein and boiling did not change the behavior of the truncated variant. Together this data show that our alternative purification method does not cause any significant alterations to the structural properties of the monomeric protein, relative to the protocol used for the WT protein.

### Characterization of the Aggregation Propensity of 7–140 α-Synuclein

**FIGURE 3:**
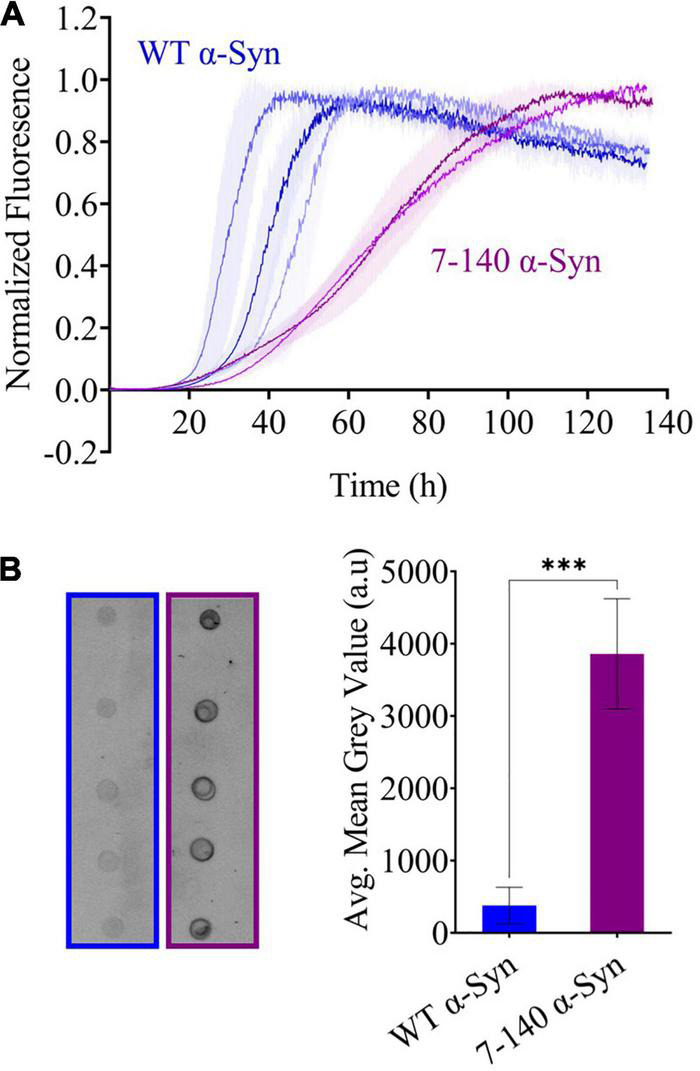
**(A)** Beaded aggregations of WT (blue) and 7–140 (purple) α-syn. WT and 7–140 α-syn have *t*_1/2_ values of 38 ± 7 and 69.7 ± 0.7 h and *t*_*lag*_ values of 28 ± 6 and 31 ± 6 h, respectively. Each data set is the average of the normalized technical replicates (*n* ≥ 3, error bars are the standard deviation of the mean). Three independent replicates for WT α-syn and two independent replicates for 7–140 α-syn are shown. **(B)** Dot blots (left) and quantifications (right) of the soluble fractions after aggregation of WT (blue) and 7–140 (purple) α-syn after aggregation (*n* = 5, values represent means and error bars are the standard deviation, Welch’s *t*-test ^***^p = 0.0002).

**FIGURE 4:**
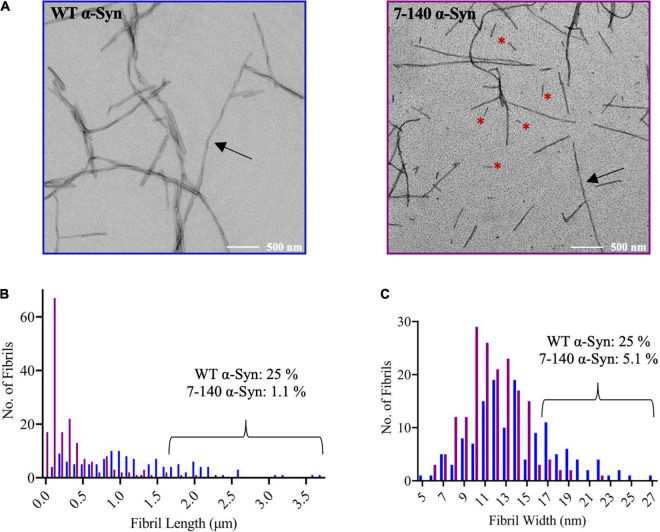
**(A)** Representative TEM images of WT and 7–140 α-syn fibrils. Arrows indicate fibrils with clear twisting, red asterisks highlight short fibrils. **(B)** Length distribution of WT (blue, *n* = 132) and 7–140 (purple, *n* = 175) α-syn fibrils. Images used for the quantification are reported in panel **(A)** and Supplementary Figure 5. The percentage of fibrils of length ≥1.7 μm (75th percentile of the length distribution of WT α-syn fibrils) is shown for each variant. **(C)** Width distribution of WT (blue, *n* = 138) and 7–140 (purple, *n* = 175) α-syn fibrils. The percentage of fibrils of width ≥17 nm (75th percentile of the width distribution of WT α-syn fibrils) is shown for each variant.

To characterize the aggregation of 7–140 α-syn, we performed ThT experiments, where 7–140 α-syn was incubated at 37°C under constant shaking in the presence of a borosilicate bead to accelerate aggregation. As a control, we analyzed WT α-syn under the same conditions (Figure 3A). We found that 7–140 α-syn aggregates at a significantly slower rate than WT α-syn. In particular, while the duration of the lag-phase (*t*_*lag*_) is the same for both proteins (∼30 h), the half-time of aggregation (*t*_1/2_) of 7–140 α-syn (∼70 h) is approximately twice as long as WT α-syn (∼40 h), suggesting that the growth phase of aggregation is affected by this truncation. Additionally, we observed that the ThT intensity at the plateau of aggregation of 7–140 α-syn is lower than that of WT α-syn (Supplementary Figure 3), suggesting that 7–140 α-syn generates a lower yield of amyloids than the WT protein. To validate this result, we compared the amounts of soluble protein at the plateau of aggregation. To do so, we collected the final timepoints of aggregation, separated the soluble and insoluble fractions by centrifugation, and performed a dot-blot analysis on the soluble fractions. We observed a significantly higher dot-blot signal for 7–140 α-syn as compared to WT α-syn, which agrees with a lower net conversion of soluble protein (Figure 3B). As a control, we also carried out a dot-blot on the monomeric proteins (Supplementary Figure 4) and show that the antibody used for these experiments can bind to 7–140 and WT α-syn monomers to the same extent, confirming that the different signal observed in Figure 3B is not due to a different antibody reactivity against the two α-syn variants. The presence of more soluble protein at the end of the aggregation for 7–140 as compared to WT α-syn suggests that the N-terminus plays a key role in the nucleation of α-syn even in absence of membranes in agreement with previous reports (Kumari et al., 2021; Yang et al., 2021). To further characterize the aggregation of 7–140 α-syn, we performed TEM on the final timepoints of aggregation and analyzed the length and width distributions of the fibrils of the two proteins (Figure 4 and Supplementary Figure 5). Our results indicate that the fibrils of 7–140 α-syn are overall shorter than those of WT α-syn, as shown by the fact that 25th and 75th percentiles of the length distributions are 0.1/0.4 and 0.6/1.7 μm for 7–140 and WT α-syn, respectively. This is further supported by the fact that only ∼1% of the fibrils of 7–140 α-syn have a length which is equal to or greater than 1.7 μm, i.e., the 75th percentile of the length distribution of WT α-syn fibrils. In terms of width, we observed that the two variants have comparable distributions, having 25th and 75th percentiles equal to 11/14 and 10/17 nm for 7–140 and WT α-syn, respectively. However, we found that only ∼5% of the fibrils of 7–140 α-syn have a width equal to or greater than 17 nm, i.e., the 75th percentile of the width distribution of WT α-syn fibrils, suggesting that 7–140 α-syn cannot efficiently form fibrils of large thickness as compared to WT α-syn.

**FIGURE 5:**
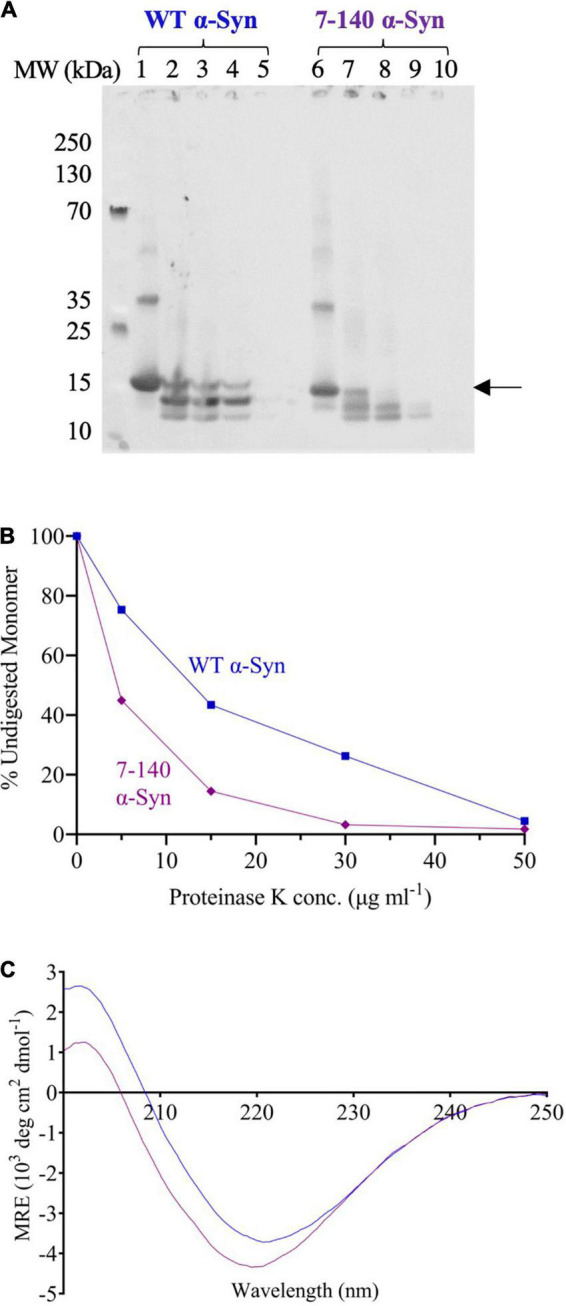
**(A)** Proteinase K digestion of WT (lanes 1–5) and 7–140 (lanes 6–10) α-syn fibrils incubated with 0, 5, 15, 30, and 50 μg/ml proteinase K, respectively. The arrow indicates the band that has been quantified and plotted. A representative experiment is shown. **(B)** The percentage of undigested monomeric WT (blue) and 7–140 (purple) α-syn at various protease concentrations. **(C)** CD spectra of WT (blue) and 7–140 (purple) α-syn fibrils. A representative spectrum for each variant is shown.

To determine if this increased fragmentation was associated to a lower fibril stability, the endpoints of aggregation were digested with proteinase K (Figures 5A,B). We found that 7–140 α-syn fibrils are more susceptible to proteinase K digestion than the WT fibrils. In particular, we observed that the amount of undigested monomeric 7–140 α-syn decreases more rapidly than that of WT α-syn as a function of the protease concentration (Figure 5B). Finally, we performed CD spectroscopy to assess the secondary structure of the fibrils and found that the spectrum of 7–140 α-syn fibrils is compatible with a higher random coil content with respect to the WT α-syn fibrils (Figure 5C).

## Conclusion

The aggregation of α-syn into amyloids is the hallmark of several neurodegenerative diseases including PD (Spillantini et al., 1997). This process is highly complex because of the numerous α-syn PTMs, including N-terminal truncation (Liu et al., 2005; Anderson et al., 2006; Dufty et al., 2007; Kellie et al., 2014). So far, the recombinant production of N-terminally truncated α-syn has been limited to protein variants starting with a Gly, retained after TEV cleavage, or a non-native Met for initiating translation. In this paper, we have described a facile method for the expression and purification of potentially any relevant N-terminally truncated fragment of α-syn without the need for the introduction of non-native residues or the use of a protease.

Our method allowed us to characterize the role of the first six N-terminal residues of α-syn in the amyloid aggregation of the protein. In our experimental conditions, we found that truncation of this region affects the aggregation of the protein and the stability of the amyloids (Figures 3, 4). Our findings are in agreement with previous reports which found that the deletion of residues 2–11 of α-syn significantly delays aggregation (Lorenzen et al., 2014), impairs membrane binding, and decreases toxicity in yeast (Vamvaca et al., 2009). We expect our method to be applied to generate additional N-terminally truncated α-syn variants to shed light into the mechanisms of the N-terminal region of α-syn.

## Data Availability Statement

The original contributions presented in the study are included in the article/Supplementary Material, further inquiries can be directed to the corresponding author.

## Author Contributions

RJT and DMV performed the experiments. RJT and FAA conceptualized the work. All authors analyzed the data and wrote the manuscript.

## Conflict of Interest

The authors declare that the research was conducted in the absence of any commercial or financial relationships that could be construed as a potential conflict of interest.

## Publisher’s Note

All claims expressed in this article are solely those of the authors and do not necessarily represent those of their affiliated organizations, or those of the publisher, the editors and the reviewers. Any product that may be evaluated in this article, or claim that may be made by its manufacturer, is not guaranteed or endorsed by the publisher.

## Abbreviations

α -syn: α -synuclein
PD: Parkinson’s disease
LB: Lewy bodies
NAC: non-amyloid- β component
LLPS: liquid-liquid phase separation
PTM: post-translational modification
WT: wild-type
*E. coli*: *Escherichia coli*
TEV: tobacco etch virus
HEPES: 4-(2-hydroxyethyl)-1-piperazineethanesulfonic acid
EDTA: ethylenediaminetetraacetic acid
CV: column volume
BME: β -mercaptoethanol
MBP: maltose binding protein
SEC: size-exclusion chromatography
SDS-PAGE: sodium dodecyl sulfate–polyacrylamide gel electrophoresis
ESI-MS: electrospray ionization mass spectrometry
ThT: Thioflavin T
CD: circular dichroism
MRE: mean residue ellipticity
*Sce*: *Saccharomyces cerevisiae*
VMA: vacuolar ATPase
CBD: chitin binding domain
TEM: transmission election microscopy.
