# Supplementary material for: A Facile Method to Produce N-Terminally Truncated α-Synuclein
Source: Front Neurosci. 2022 May 27;16:881480. doi: 10.3389/fnins.2022.881480 (PMC9184721; doi:10.3389/fnins.2022.881480)
Supplement: Supplementary file 1 [file Data_Sheet_1.pdf]

Supplementary Material

A Facile Method to Produce N-Terminally Truncated α-Synuclein

Supplementary Figures

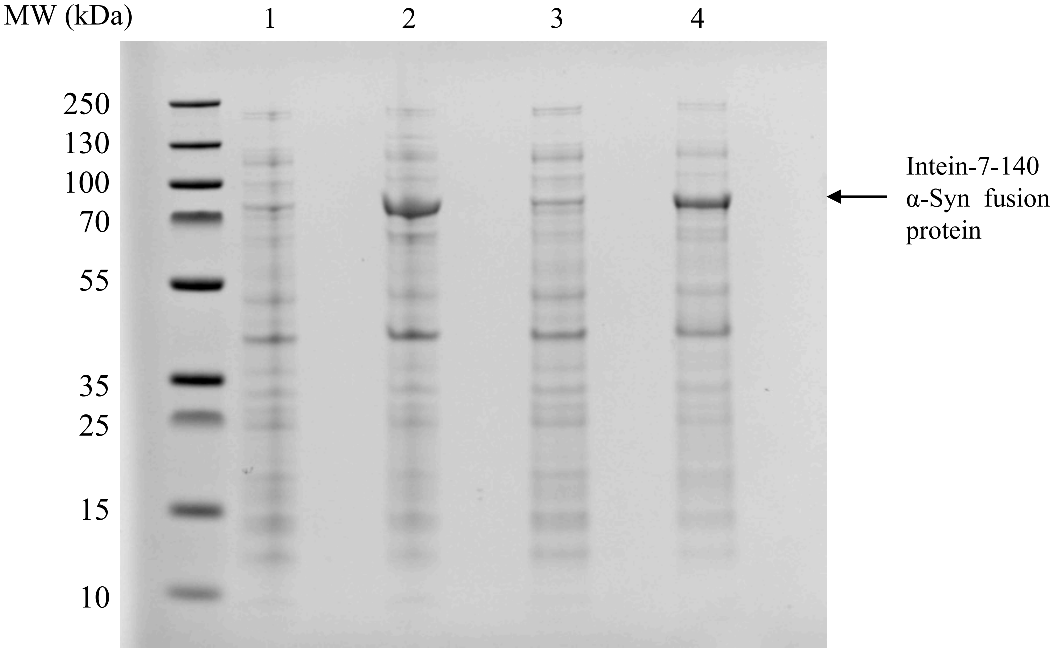

Supplementary Figure 1. SDS-PAGE of cell lysate before and after induction of protein expression with 1 mM IPTG. We induced expression at 28°C overnight (lane 2) and at 37°C for 4 hours (lane 4). Lanes 1 and 3 contain the lysate of cell aliquots taken before induction at 28°C and 37°C respectively. The indicated band in lanes 2 and 4 corresponds to the 72.1 kDa intein-7-140 α-syn fusion protein.

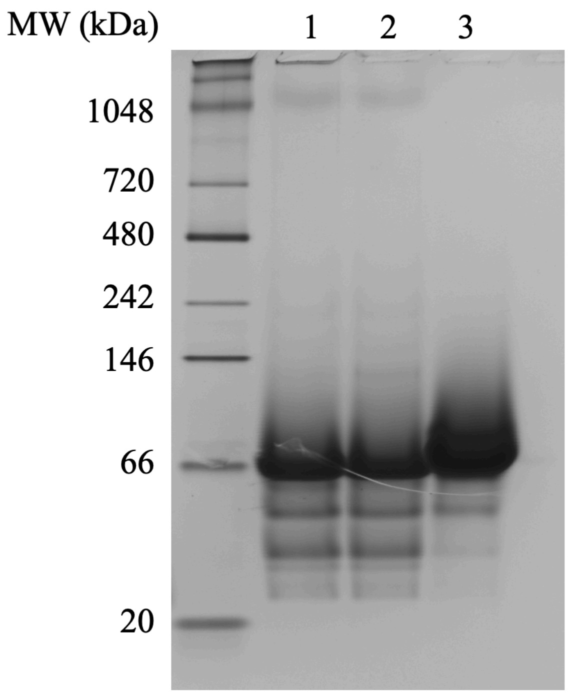

Supplementary Figure 2. Native PAGE of 7-140 α-syn before (lane 2) and after (lane 1) boiling at 80°C for 20 mins, alongside conventionally purified WT α-syn (lane 3).

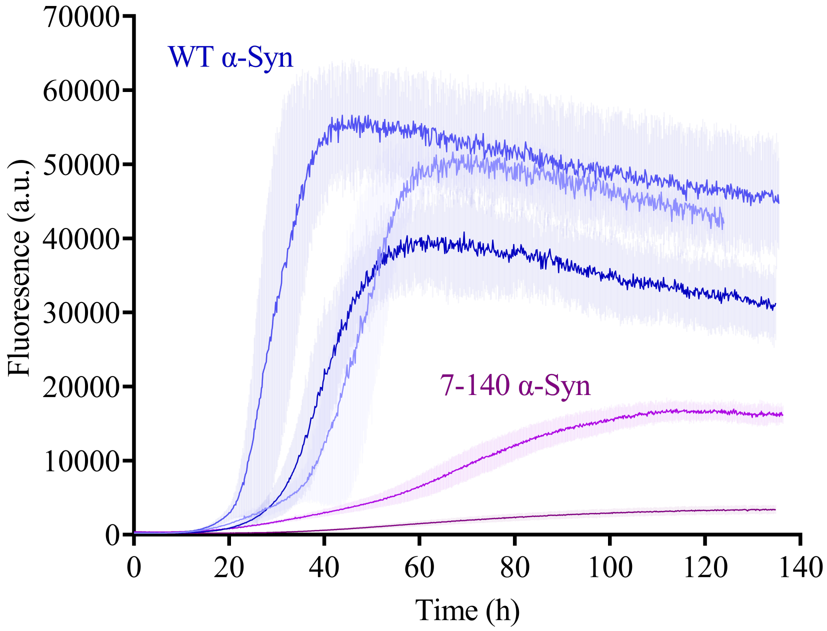

Supplementary Figure 3. Raw data for the beaded aggregation of WT α-syn (blue) and 7-140 α-syn (purple). Error bars representing the standard deviation are shown as transparent bars. Each data set is the average of at least three technical replicates.

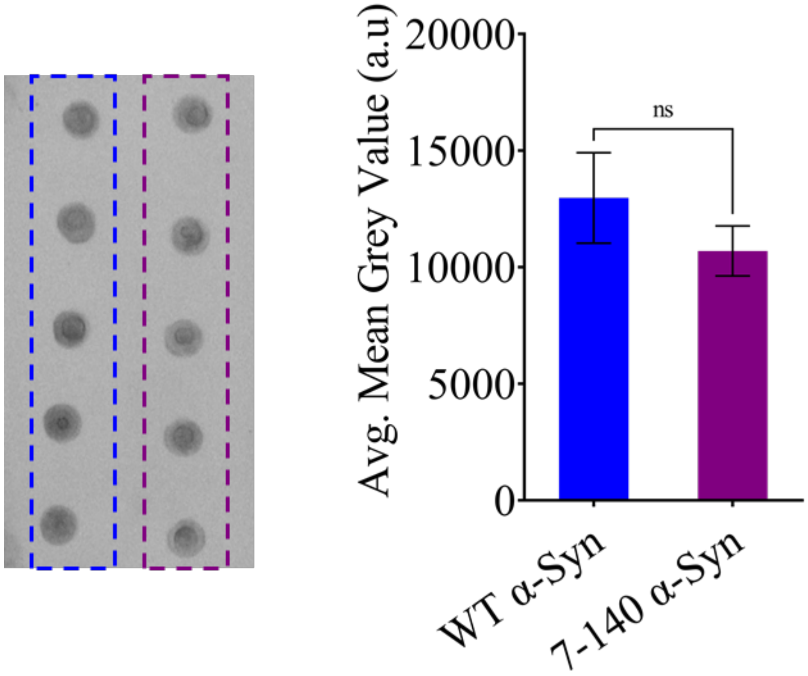

Supplementary Figure 4. Dot blot (left) and corresponding quantification (right) of monomeric WT (blue) and 7-140 (purple) α-syn ($n = 5$, values represent means and error bars are the standard deviation, Welch's *t*-test ns, $p = 0.0597$).

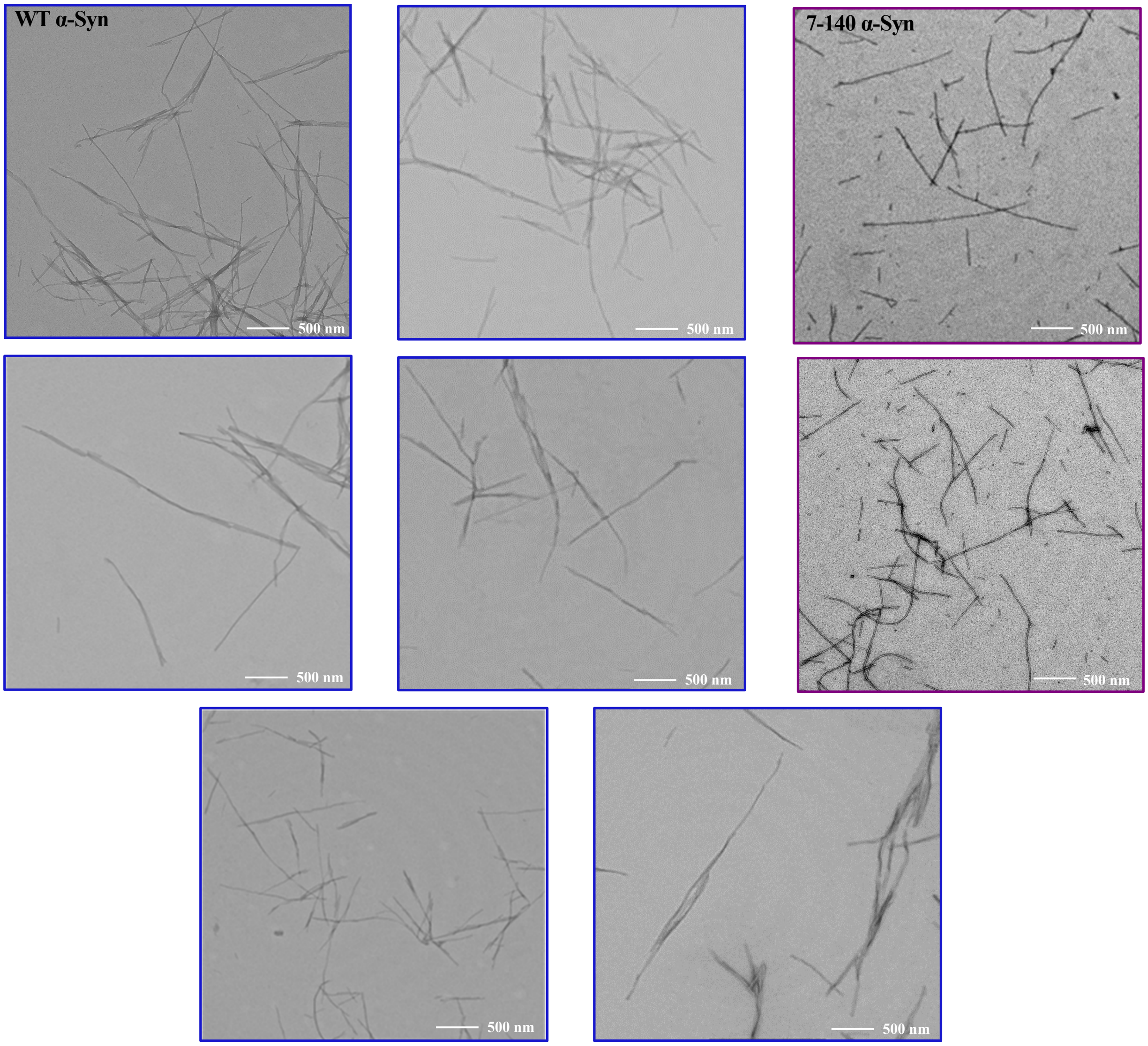

Supplementary Figure 5. Additional TEM images of WT (blue) and 7-140 (purple) α-syn fibrils after ~140 h of aggregation that have been used for the quantification of fibril length and width presented in Figure 4B and 4C.
